# Effect of Resistive Load on the Inspiratory Work and Power of Breathing during Exertion

**DOI:** 10.1371/journal.pone.0049681

**Published:** 2012-11-27

**Authors:** Thomas Powell, Edgar Mark Williams

**Affiliations:** Faculty of Health, Sport and Science, University of Glamorgan, Pontypridd, United Kingdom; Universidad Europea de Madrid, Spain

## Abstract

The resistive work of breathing against an external load during inspiration (WR_I_) was measured at the mouth, during sub-maximal exercise in healthy participants. This measure (which excludes the elastic work component) allows the relationship between resistive work and power, ventilation and exercise modality to be explored. A total of 45 adult participants with healthy lung function took part in a series of exercise protocols, in which the relationship between WR_I_, power of breathing, PR_I_ and minute ventilation, 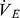 were assessed during rest, while treadmill walking or ergometer cycling, over a range of exercise intensities (up to 150 Watts) and ventilation rates (up to 48 L min^−1^) with applied constant resistive loads of 0.75 and 1.5 kPa.L.sec^−1^. Resting WR_I_ was 0.12 JL^−1^ and PR_I_ was 0.9 W. At each resistive load, independent of the breathing pattern or exercise mode, the WR_I_ increased in a linear fashion at 20 mJ per litre of 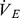, while PR_I_ increased exponentially. With increasing resistive load the work and power at any given 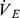 increased exponentially. Calculation of the power to work ratio during loaded breathing suggests that loads above 1.5 kPa.L.sec^−1^ make the work of resistive breathing become inhibitive at even a moderate 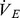 (>30 L sec^−1^). The relationship between work done and power generated while breathing against resistive loads is independent of the exercise mode (cycling or walking) and that ventilation is limited by the work required to breathe, rather than an inability to maintain or generate power.

## Introduction

Resistive loads are used to train respiratory muscles in athletes and in rehabilitation of people with pulmonary disease or spinal injuries [1]–[3]. Other studies use external resistive load detection as a tool to study dyspnoea sensitivity [4]. Resistive work is dependent on ventilation, and different exercise modes, for example walking and cycling place different metabolic demands on the body. A measure of the work performed during breathing can be derived from the product of the volume and pressure change generated during the respiratory cycle. The mechanical work of breathing which includes the elastic and resistive components, defined per litre of ventilation in resting healthy subjects is around 0.35±0.1 JL^−1^ while the power generated is around 2.4±0.7 W [5]. The power generated is thus influenced by respiratory rate and volume of air moved. Measuring these parameters often involve using invasive techniques and require an estimate of chest wall compliance [6].

At rest the inspiratory work of breathing is consistently larger than expiratory work. However this changes with exercise when expiration also becomes an active process. Measuring work only during the inspiratory phase of the respiratory cycle provides a simple index of the muscle-driven work irrespective of the expiratory phase.

This study aims to use a new non-invasive method to measure the resistive work of breathing and the subsequent power generated (WR_I_ and PR_I_ respectively) while breathing against added resistive loads and explore this relationship with ventilation during sub-maximal exercise, sitting, walking or cycling. This study while observational is unique in that the loading was applied in “real-time” using a servo-controlled variable orifice pneumotachograph allowing both resistive inspiratory work and power to be assessed simultaneously.

## Methods

### Study particpants

Forty five healthy participants (Table 1) gave written informed consent and completed a good health screening questionnaire before taking part. All studies were approved by the Faculty of Health, Sport and Science, University of Glamorgan, Ethics Committee.

**Table 1:** Participant demographics for each exercise level.

	Level 1N = 10	Level 2N = 20	Level 3N = 15
**Sex (M;F)**	6∶4	12∶8	15∶0
**Age (yrs)**	25–64	20–48	18–29
**FEV_1_%predicted** *	105±14	98±9	108±13
**MIP (cm H_2_O)** *	112±5	108±36	124±34

The mean ±SD is shown.

* No significant differences between groups, p>0.05 with all participants having healthy lung function.

Respiratory function was measured using a standard pneumotachograph which was modified to include an extra computer controlled variable orifice fitted into the mouthpeice (MicroRMA, MicroMedical Ltd, Kent, UK). In addition to measuring flow (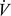) and mouth pressure (P), the device was able to adjust airflow resistance by altering the internal diameter of the flow head (making P/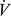 constant), using a servo controlled orifice adjusted via feedback at 100 Hz. A constant resisitve load, R_K_ could be thus be maintianed throughout the respiratory cycle. The device was calibrated daily for flow using a 3 litre syringe and pressure by applying a 10 cmH_2_0 pressure using a water U-tube manometer. When the orifice was fully open and no bacterial filter added the resitance of the pneumotachograph was 0.14 kPa.L. sec^−1^ at a flow of 1 Lmin^−1^.

### Protocol

After gaining consent, body weight and height were measured and baseline spirometry performed including the measurement of maximal inspiratory pressure (MicroRPM, Micro Medical Ltd, Kent, UK) [7]. Each trial, whether seated or exercising required the participant to breathe through the mouthpiece for six minutes with an applied resistive (R_K_) load. All subjects wore nose clips and had no prior experience of breathing through the device. When a participant indicated they were ready to begin, they were asked to breathe through the hand-held flow-head connected to a mouthpiece and bacterial filter until instructed to stop. The trial ended after six minutes or prematurely if they gave up or failed to maintain a tight seal around the mouthpiece. Participants had at least ten minutes rest between each trial or until they felt ready to continue. All 45 participants were assessed with no added resistance.

### Exercise Level One

This pilot stage of the study was designed to establish the difference in WR_I_ and PR_I_ between sedentary and exercising participants with no added resistive load. Ten participants were asked to complete two trials with the variable orifice open providing no additional resistance (R_K_ = 0 kPa.L.sec^−1^) once while seated and again while walking on a treadmill (LE 200 CE, Viasys Healthcare Ltd, UK) at 4 km.h^−1^ (equivalent to 31.5 Watts). Without added load, the main resistance experienced by the participants is that already provided by their existing intrinsic airway resistance, which is approximately 0.2–0.3 kPa.L.sec^−1^ in healthy individuals. In this study where no extra resistance has been added a value of 0.25 kPa.L.sec^−1^ has been used.

### Exercise Level Two

Level two was designed to build on the exercise used at level one and establish the effect of adding resistive loads, to change the exercise modality and control the respiratory rate. Another twenty participants completed four trials, two seated and two exercise trials at constant resistive loads, R_K_ of 0.75 and 1.5 kPa.L.sec^−1^, loads selected to provide a mild and heavy load respectively. Breathing rate was paced using an electronic metronome set at 15 breaths.min^−1^with a duty cycle of 0.5. Ten participants completed exercise tests on the treadmill at 4 km.h^−1^ (level) and the remaining ten completed exercise tests on a computer controlled cycle ergometer (ViaSprint, Viasys Healthcare Ltd, UK) at 50 Watts.

### Exercise Level Three

Level three was used to extend the range of 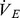. Fifteen participants completed five self-paced breathing trials, three seated at added loads of 0, 0.75 and 1.5 kPa.L.sec^−1^ and two cycling trials at 100 and 150 W with an R_K_ load of 0.75 kPa.L.sec^−1^. Participants were subsequently selected for the 150 W trial if their maximum heart rate was ≤130 min^−1^ during the previous 100 W trial, this reflected 2/3 of their maximum predicted heart rate indicating that they would be able to complete the 150 W trial safely.

### Study Measures

Throughout each six-minute trial, flow and pressure were sampled at 100 Hz. This allowed post-test analysis of the recorded data and division into periods of inspiration and expiration, with a change in mouth pressure from negative to positive denoting the end of inspiration and start of expiration. As all participants were in a steady breathing state when tested the mean values for the six-minute period were used.

The inspiratory ventilatory resistive work was calculated as WR _I_ = ∫ Mouth pressure x volume and calculated for each inspired tidal volume and expressed in JL^−1^. The power generated was calculated as WR_I_×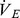 and is expressed in Watts (J.min^−1^) [6], [8], [9].

### Statistical Analysis

Regression techniques were used to assess the relationship between parameters and derived using statistical software (Sigmaplot V12, Systat, UK). The coefficient of predictive power, P^2^ was calculated according to the method of Mediavilla *et al.* [10]. The level of significance was p<0.05.

## Results

The 45 participants tolerated all the seated resting trials (n = 105) with none ending early. At rest in 16 participants (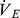 7.4±0.9, mean ± SD), WR_I_ was 0.12±0.03 JL^−1^, while PR_I_ was 0.86±0.28 W at rest providing smaller values reported in other studies [5], [11]–[13].

During exercise, (level 2) four trials were not completed, in three cases because participants could not maintain the paced ventilation rates through the device and in one case due to technical equipment failure. All 100 W exercise trials (level 3) were successfully completed, eight of these participants having a heart rate of ≤130 min^−1^, were recruited to the 150 W cycle ergometry trial.

**Figure 1:**
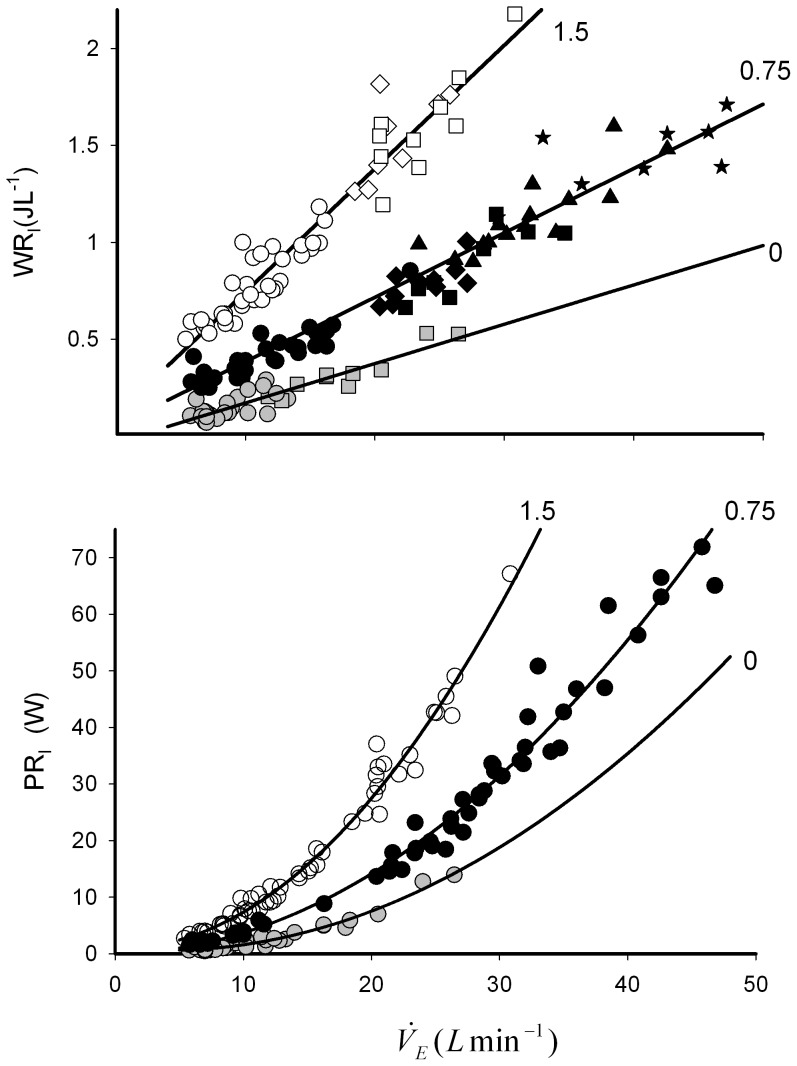
Relationship between ventilation (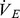) and the inspiratory work to overcome resistance at the mouth (WR_I_) (Upper panel) and the associated inspiratory power (PR_I_) (lower panel) with increasing resistive load (R_K_). For sake of clarity exercise mode has been omitted on the lower panel (see text) The applied loads were 0 (grey fill), 0.75 (black fill) and 1.5 (no fill) kPa.L.sec^−1^. In the upper panel, exercise modes; **○**, resting and seated; □, walking at 4 kmh^−1^; ◊, cycling at 50 watts; Δ, cycling at 100 watts; ★stars, cycling at 150 watts. Fitted linear regression are shown for each applied load, R^2^ = 0.85, 0.93, 0.93, and P^2^ = 0.83,0.93, 0.92 at 0, 0.75, 1.5 kPa.L.sec^−1^respectively. In the lower panel no distinction is made between exercise mode and fitted quadratic lines are shown for each applied load, at 0, Y = 0.98+−0.2^×^+0.03^×2^, R^2^ = 0.97 P^2^ = 0.996, at 0.75 kPa.L.sec^−1^, Y = 0.05+0.025^×^+0.034^×2^, R^2^ = 0.97, P^2^ = 0.97, and at load 1.5 kPa.L.sec^−1^ Y = 0.94+−0.043^×^+0.07^×2^ R^2^ = 0.98, P^2^ = 0.98. In all cases p<0.001.

Irrespective of the breathing pattern or exercise modality, minute ventilation, 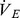 (from rest to around 48 L min^−1^) was seen to increase with WR_I_ in a linear fashion (Fig. 1 Upper panel). The slope of this relationship was steeper as the load increased, from 0.02 (R^2^ 0.85, P^2^ 0.83 and p<0.001) with no added load to 0.033(R^2^ 0.93, P^2^ 0.93 and p<0.001), and 0.064 (R^2^ 0.93, P^2^ 0.92 and p<0.001) J for each litre.min^−1^ increase in 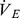 at added loads of 0.75 and 1, 5 kPaLsec^−1^ respectively. The associated resistive inspiratory power of breathing, POB_I_ increased exponentially with 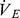 with the rate of change increasing with added load (Fig. 1, Lower panel). With unloaded breathing the non-linear regression values of fit were R^2^ 0.97, P^2^ 0.996, P<0.001, for a load of 0.75 kPaLsec^−1^ R^2^ 0.97, P^2^ 0.97, p<0.001 and for the greatest load, R^2^ 0.98, P^2^ 0.98 and p<0.001, (Figure 1, lower panel).

**Figure 2:**
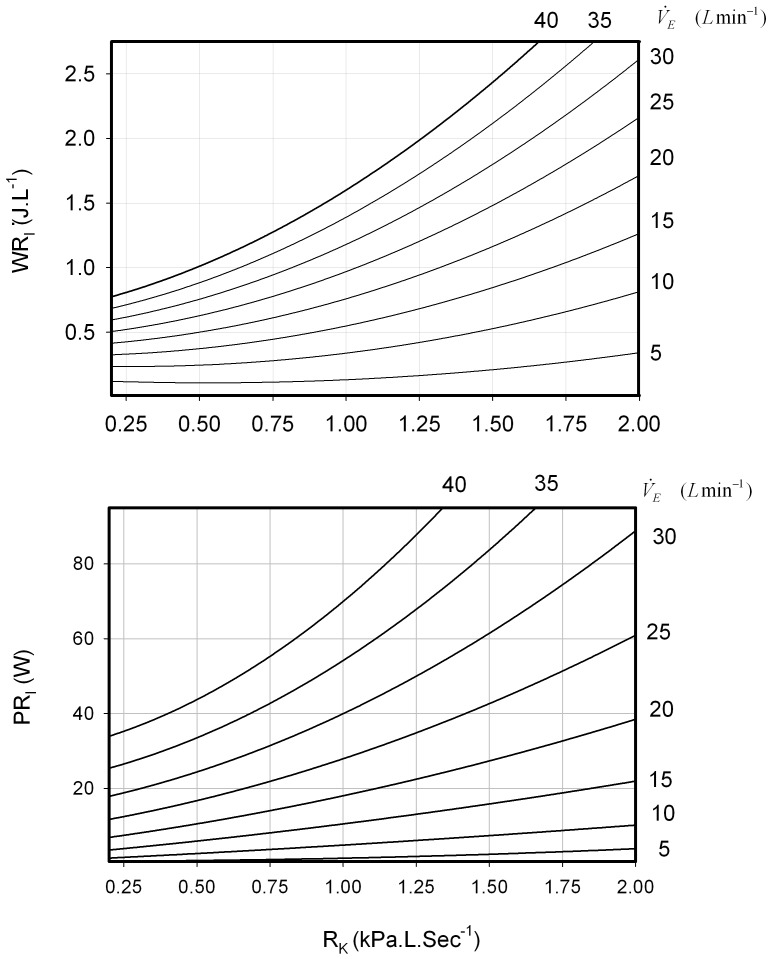
Relationship between R_K_ (kPa.L.sec^−1^) WR_I_ (JL^−1^) (Upper panel) and PR_I_ (J min^−1^) (Lower panel) at increasing 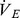 (L min^−1^). See text for details.

**Figure 3:**
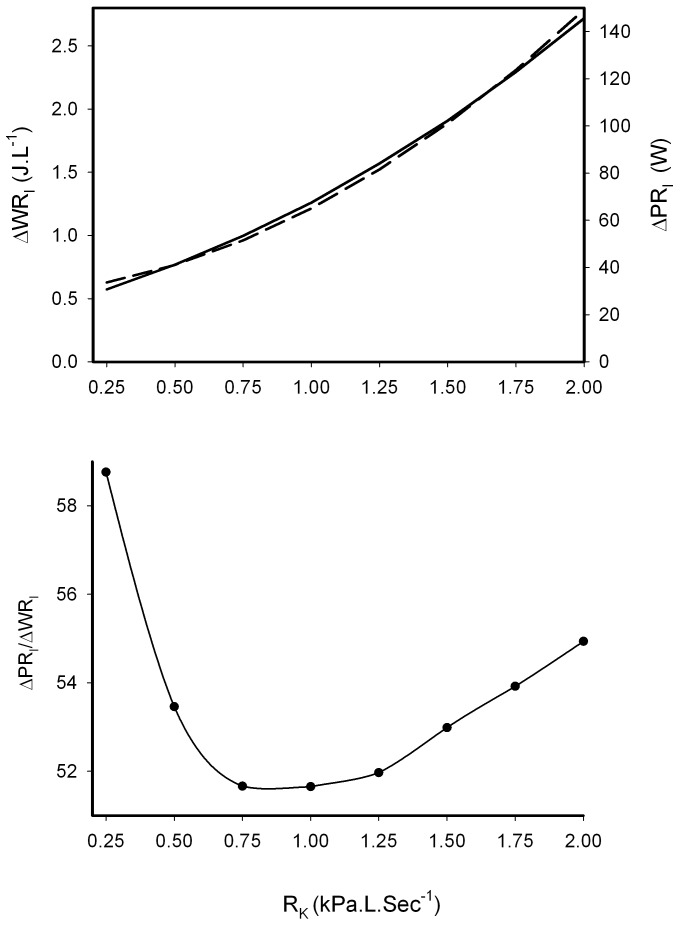
The calculated change in the resistive work (–) and power (—) of breathing of inspiration after increasing ventilation, 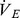 from 10 to 40 L.min^−1^(Upper panel). The lower panel shows calculated change in the power to work ratio with applied load after increasing 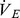 from 10 to 40 L.min^−1^.

For specific ventilation rates the WR_I_ and PR_I_ values were calculated at each of the three levels of resistance, these three values were plotted and fitted with regression equations to construct isopleths for each five litre increase in ventilation for WR_I_ and PR_I_ from 5 to 40 L min^−1^(Fig. 2). These values allowed the calculation in the change in resistive work and power increase with an increase in 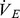 from 10 to 40 L min^−1^ (Fig. 3, upper panel). Under these conditions the power to work ratio changes with resistive load, the ratio being lowest between 0.75 and 1.25 kPa.L.min^−1^ (Fig. 3 lower panel). No relationship (p>0.05) between respiratory muscle strength as measured using MIP and the resting WR_I_ (No added R_k_) was observed (Table 1).

## Discussion

The study describes a method for assessing resistive work and power during inspiration during sub-maximal exercise while either walking or cycling and shows that while WR_I_ increases linearly with ventilation rate (over 5–48 L min^−1^), the associated power output increases exponentially (Fig. 1). At any particular 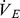, the addition of a resistive load increases the ventilatory work in an exponential manner along with power (Fig. 2). The range at which the power to work ratio is lowest is between 0.75 and 1.25 kPa.L.min^−1^(Fig. 3).

Measuring work at the mouth only measures the work required overcoming airway resistance, but the majority of the work of breathing (around 60–70%) is done overcoming elastic recoil of the lungs and chest wall [8]. Ventilation and breathing pattern influence elastic work and power, with elastic work being minimized by shallow rapid breathing [14]. In disease states such as COPD, minimising this elastic work alters breathing patterns and static lung volumes. However, in exercise, ventilatory drive is more important in determining the breathing pattern and ventilation rate. With exercise, breathing is rapid, but not shallow, as tidal volume soon reaches a maximum [15]. Under these conditions elastic and mechanical work are likely to be maximised [16].

When breathing rate was self-paced all the participants were able to complete all the six-minute trials even while exercising at intensities up to 150 W, and loads of 1.5 kPa.L.sec^−1^. However when pacing was imposed, three trials were halted early (exercise level 2) due to the participants being unable to maintain the breathing rate of 15 min^−1^. When the breathing was unpaced the technique was easily tolerated by the participants as this allowed the participants to meet ventilatory demand in two ways, via altering breathing rate or tidal volume. Pacing is likely to become problematic at higher levels of aerobic exercise as increases in ventilation can only be met by raising the breathing rate as tidal volume reaches a plateau typically between 2–3 L [15]. While a paced breathing rate would help standardize the test procedure, this is not practical during exercise.

With an oral resistive load added, ventilation, 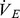 was unchanged within each exercise modality, however, the work increased with exercise intensity (Fig. 1). An increase in ventilation is matched by an increase in WR_I_. Thus irrespective of the ventilatory driver (i.e. exercise) the WR_I_ is just dependent upon the volume of air required (Fig. 1) [13]. This relationship between ventilation and WR_I_ is linear, which shows that at a given resistive load, the WR_I_ remains constant i.e. at 0.75 kPa.L.min^−1^ each litre of extra ventilation requires 33 mJ of work (Fig. 1). Although this increase is small it is the total work that makes breathing unsustainable at higher ventilation rates and resistive loads.

The power output increases exponentially with ventilation rate (Fig. 1), the ability to generate this power has been shown to be unaffected by fitness levels and high altitude [9], [12], [17]. Other studies have shown power is gender dependent [18], [19].

The addition of the mid-range load of 0.75 kPa.L.sec^−1^ was tolerated well at the sub-maximal exercise levels used. This load in seated trials elicited a significant response in WR_I_ in our healthy individuals, from 0.14 with no added resistance to 0.38 JL^−1^ (Fig. 1). At higher loads the WR_I_ soon becomes overpowering and few subjects felt comfortable exercising while their breathing was restricted in this way. The upper limit to a tolerable resistive load during exercise before fatigue or task failure occurs was not measured directly but our data would suggest that an R_k_ load of 2 kPa.L.sec^−1^, the WR_I_ required to generate sufficient pressure to ventilate the lungs would become inhibitive at even moderate ventilation rates (>30 L sec^−1^) (Fig. 2). Whether the ability to overcome a high R_K_ load is dependent on fitness levels (and motivation) or dependent upon inherent respiratory function or a mixture of both has yet to be investigated. The effect of increased resistive load on exercise is important in individuals with increased intrinsic airway resistance such as asthmatics, as even small increases in airway resistance would appear to result in large increases in work and power generation which would alter their endurance capabilities (Fig. 2). In patients with chronic airways obstruction the matter is more complex as there are increases in elastic and threshold loads as well as resistive loads due to the breathing frequency dependency of compliance plus the presence of dynamic hyperinflation.

At any given ventilation rate the effect of increasing the resistive load, R_K_ alters the power to work ratio. Fig. 3 shows how this relationship changes when 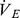 increases, four-fold (from 10 to 40 L min^−1^), in this example. This shows that the system's power to work ratio, changes with the addition of a resistive load and is at its best between 0.75 and 1.25 kPa.L.min^−1^ (Fig. 3). After this the power to work ratio increases and could reflect an exercised-induced change in the end expiratory lung volume [20]. This predicts that at higher resistance breathing should become relatively easier which is clearly not the case. The predicted WR_I_ at 2 kPaLmin^−1^, even at a low 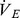 is high (2.7 JL^−1^ 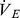 40 L.min^−1^, Fig. 2), at maximal levels of exercise with no added resistance and a high 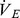 (around 175 L min^−1^); WR_I_ rarely goes higher than 2.2 JL^−1^ [18]. At this high work rate of 2.7 JL^−1^, power output may be improved, but it seems that the respiratory system is unable to maintain this work rate for long. The ability to breathe against a load is thus restricted by the ability to do mechanical work rather than the power requirement or generation. This may be different in people with obstructed airways, who exhibit rapid shallow breathing during exercise and then power generation may be more affected than work because of the high breathing frequency.

The efficacy of respiratory training devices that apply respiratory loads are often evaluated on how they increase respiratory muscle strength, but the focus should be on improvements in respiratory endurance, as this would provide a better measure of the ability to tolerate increased work of breathing. The device used here provides a simple, non-invasive method for applying a constant restive load to induce a steady- state of resistive work when exercising, an advantage over invasive techniques.

Though in healthy humans there are only a few occasions when the reserve of respiratory muscle function is utilised sufficiently to cause fatigue, there are certain groups of patients where the endurance properties of the respiratory muscles may be critical to survival or necessary for adequate exercise tolerance [21]. With the increased use of pulmonary rehabilitation programmes it is necessary to monitor endurance performance rather than strength; valid measures of endurance are thus needed to assess the outcomes of such interventions [22]. In the current study no relationship was found between respiratory muscle strength and work and power output, muscle strength is often muted as a surrogate of respiratory endurance. If strength and endurance were related then a strong link should exist. However caution is necessary as muscle strength under isometric conditions (as with MIP) is different in muscles undergoing rapid movement as in breathing [8].

In conclusion, this study shows that resistive inspiratory work and power change with ventilation rate, and applied load. While exertion serves to increase 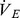, the exercise mode does not influence resistive work or power output. At any given ventilation rate the work done and power output increases exponentially with added resistance, with the power to work ratio, the mechanical advantage, is at its best between 0.75 and 1.25 kPa.L.min^−1^. This exponential relationship ensures that at high ventilation rates, mechanical work limits breathing, through an inability to inspire, rather than a lack of ability to maintain ventilation (power). Thus in the respiratory system, inspiratory muscle strength is more important than endurance in limiting ventilation.
